# Supplementary material for: Pyruvate Dehydrogenase Kinase 1 inhibition mediated oxidative phosphorylation enhancement in cartilage promotes osteoarthritis progression
Source: BMC Musculoskelet Disord. 2023 Jul 20;24:597. doi: 10.1186/s12891-023-06585-6 (PMC10357736; doi:10.1186/s12891-023-06585-6)
Supplement: Supplementary file 1 — Additional file 1. [file 12891_2023_6585_MOESM1_ESM.zip › KEGG.Gsea.1653622432464/KEGG_BIOSYNTHESIS_OF_UNSATURATED_FATTY_ACIDS.html]

Details for gene set KEGG\_BIOSYNTHESIS\_OF\_UNSATURATED\_FATTY\_ACIDS[GSEA]

|  || Dataset | 55457\_collapsed\_to\_symbols.cls.cls #OA\_versus\_normal.cls.cls #OA\_versus\_normal\_repos |
| Phenotype | cls.cls#OA\_versus\_normal\_repos |
| Upregulated in class | OA |
| GeneSet | KEGG\_BIOSYNTHESIS\_OF\_UNSATURATED\_FATTY\_ACIDS |
| Enrichment Score (ES) | 0.2865993 |
| Normalized Enrichment Score (NES) | 0.9377129 |
| Nominal p-value | 0.5462963 |
| FDR q-value | 0.73491037 |
| FWER p-Value | 1.0 |
Table: GSEA Results Summary

  

Fig 1: Enrichment plot: KEGG\_BIOSYNTHESIS\_OF\_UNSATURATED\_FATTY\_ACIDS      
 Profile of the Running ES Score & Positions of GeneSet Members on the Rank Ordered List

  

| SYMBOL | TITLE | RANK IN GENE LIST | RANK METRIC SCORE | RUNNING ES | CORE ENRICHMENT || 1 | HSD17B12 | hydroxysteroid 17-beta dehydrogenase 12 [Source:HGNC Symbol;Acc:HGNC:18646] | 795 | 0.570 | 0.0426 | Yes |
| 2 | ACOT7 | acyl-CoA thioesterase 7 [Source:HGNC Symbol;Acc:HGNC:24157] | 971 | 0.524 | 0.1249 | Yes |
| 3 | TECR | "trans-2,3-enoyl-CoA reductase [Source:HGNC Symbol;Acc:HGNC:4551]" | 1047 | 0.508 | 0.2120 | Yes |
| 4 | ACOX1 | acyl-CoA oxidase 1 [Source:HGNC Symbol;Acc:HGNC:119] | 1401 | 0.443 | 0.2655 | Yes |
| 5 | PECR | peroxisomal trans-2-enoyl-CoA reductase [Source:HGNC Symbol;Acc:HGNC:18281] | 1985 | 0.363 | 0.2866 | Yes |
| 6 | ELOVL6 | ELOVL fatty acid elongase 6 [Source:HGNC Symbol;Acc:HGNC:15829] | 3253 | 0.226 | 0.2295 | No |
| 7 | ACOT2 | acyl-CoA thioesterase 2 [Source:HGNC Symbol;Acc:HGNC:18431] | 4046 | 0.152 | 0.1958 | No |
| 8 | HADHA | hydroxyacyl-CoA dehydrogenase trifunctional multienzyme complex subunit alpha [Source:HGNC Symbol;Acc:HGNC:4801] | 4163 | 0.141 | 0.2126 | No |
| 9 | FADS2 | fatty acid desaturase 2 [Source:HGNC Symbol;Acc:HGNC:3575] | 5025 | 0.071 | 0.1588 | No |
| 10 | ACAA1 | acetyl-CoA acyltransferase 1 [Source:HGNC Symbol;Acc:HGNC:82] | 5175 | 0.058 | 0.1578 | No |
| 11 | HACD1 | 3-hydroxyacyl-CoA dehydratase 1 [Source:HGNC Symbol;Acc:HGNC:9639] | 5342 | 0.044 | 0.1530 | No |
| 12 | YOD1 | YOD1 deubiquitinase [Source:HGNC Symbol;Acc:HGNC:25035] | 5582 | 0.026 | 0.1392 | No |
| 13 | HACD2 | 3-hydroxyacyl-CoA dehydratase 2 [Source:HGNC Symbol;Acc:HGNC:9640] | 6162 | -0.017 | 0.0974 | No |
| 14 | ACOX3 | "acyl-CoA oxidase 3, pristanoyl [Source:HGNC Symbol;Acc:HGNC:121]" | 6747 | -0.059 | 0.0628 | No |
| 15 | FADS1 | fatty acid desaturase 1 [Source:HGNC Symbol;Acc:HGNC:3574] | 8079 | -0.161 | -0.0110 | No |
| 16 | ELOVL2 | ELOVL fatty acid elongase 2 [Source:HGNC Symbol;Acc:HGNC:14416] | 8080 | -0.161 | 0.0185 | No |
| 17 | SCD | stearoyl-CoA desaturase [Source:HGNC Symbol;Acc:HGNC:10571] | 9824 | -0.303 | -0.0613 | No |
| 18 | BAAT | bile acid-CoA:amino acid N-acyltransferase [Source:HGNC Symbol;Acc:HGNC:932] | 10523 | -0.376 | -0.0467 | No |
| 19 | ELOVL5 | ELOVL fatty acid elongase 5 [Source:HGNC Symbol;Acc:HGNC:21308] | 11272 | -0.467 | -0.0193 | No |
| 20 | SCD5 | stearoyl-CoA desaturase 5 [Source:HGNC Symbol;Acc:HGNC:21088] | 12597 | -0.796 | 0.0235 | No |
Table: GSEA details [plain text format]

  

Fig 2: KEGG\_BIOSYNTHESIS\_OF\_UNSATURATED\_FATTY\_ACIDS      
 Blue-Pink O' Gram in the Space of the Analyzed GeneSet

  

Fig 3: KEGG\_BIOSYNTHESIS\_OF\_UNSATURATED\_FATTY\_ACIDS: Random ES distribution      
 Gene set null distribution of ES for **KEGG\_BIOSYNTHESIS\_OF\_UNSATURATED\_FATTY\_ACIDS**
